# Isoflavonoids from *Crotalaria albida* Inhibit Adipocyte Differentiation and Lipid Accumulation in 3T3-L1 Cells via Suppression of PPAR-γ Pathway

**DOI:** 10.1371/journal.pone.0135893

**Published:** 2015-08-18

**Authors:** Qinhu Sun, Guixin Chou

**Affiliations:** 1 The MOE Key Laboratory for Standardization of Chinese Medicines, Institute of Chinese Materia Medica of Shanghai University of Traditional Chinese Medicine, Shanghai, China; 2 Shanghai R&D Center for Standardization of Chinese Medicines, Shanghai, China; National Research Council of Italy, ITALY

## Abstract

Two 2″-isopropenyl dihydrofuran isoflavonoids (**1** and **3**), one 2″-isopropenyl dihydrofuran chromone (**2**), as well as 13 known compounds were isolated from the herbs of *Crotalaria albida*. Their structures and relative configurations were elucidated via NMR and HRESIMS analyses. The 2″ S absolute configuration of **1** and **2** were deduced by comparing their NOESY spectra with that of **3**, which was determined via single crystal X-ray diffraction (CuKα). The 3R absolute configuration of **1** was determined by CD. Compounds **1**, **2**, and **3** inhibit the adipocyte differentiation and lipid accumulation of 3T3-L1 through down-regulation of PPAR-γ activity.

## Introduction

*Crotalaria albida* Heyne ex Roth is a member of the subfamily Papilionoideae within Fabaceae and is a shrubby perennial herbs mainly distributed in the Southeast provinces of China[1]. In our previous research, we reported two pyrrolizidine alkaloids[2] isolated from this plant.

The isoflavonoids are an important subclass of the flavonoids and are mostly distributed in the subfamily Papilionoideae of the Fabaceae[3]. The isoflavonoids are also called phytoestrogens[4] and are cardioprotective[5], anticarcinogenic[6], antidiabetic and hypolipidemic[7]. They also prevent bone loss[8] and arteriosclerosis[9].

Peroxisome proliferators-activated receptors (PPARs) belong to one of the nuclear receptor superfamilies and control expression of a set of genes that regulate lipid and glucose metabolism[10, 11]. Of the PPARs, peroxisome proliferator-activated receptor gamma (PPAR-γ) is a ligand-activated transcription regulator of adipocyte differentiation. It has been a molecular target for combating obesity and diabetes for decades[12, 13].

A natural products search found that the PPAR-γ antagonist is an important path in new drug discovery and is involved in type 2 diabetes, obesity and other metabolic diseases[14, 15]. Currently, berberine[15], tanshinone IIA[16], mycophenolic acid[17] and some germacranolide compounds[18] that present PPAR-γ antagonism effects have been shown to inhibit adipocyte differentiation and lipid accumulation in 3T3-L1 cells, reduce fat mass and weight, improve the glucose tolerance, and ameliorate glucose and lipid metabolism in the blood and liver. Thus, they are being considered as potential drugs for the treatment of obesity and diabetes.

As a part of an ongoing research program for the discovery of natural PPAR-γ antagonists from *C*. *albida*[19], this paper focuses on the isolation, structural determination and the evaluation of PPAR-γ antagonist activity of isoflavonoids.

## Material and Methods

### General

A KRÜSS P800-T polarimeter was used to measure optical rotations. The 1D and 2D NMR spectra were obtained with Bruker AV-400 or AV-500 on 500\400 MHz for ^1^H and 125\100 MHz for ^13^C, CD_3_OD (δ_H_ 3.33; δ_C_ 49.3). The HRESIMS was performed using Waters UPLC Premier QTOF spectrometer. Prep-HPLC (Agilent 1260 Series) was performed on a C-18 column (SHISEID-PACK 20 mml × 250 mm, 5 um). The preparatory silica gels (100−300 mesh) and Sephadex LH-20 were obtained from QMC Co., and Ltd. GE-H Co., Ltd[2].

Rosiglitazone was purchased from Sigma-Aldrich Chemical Co. Fetal bovine serum (FBS) and Dulbecco’s modified Eagle Medium (DMEM) were purchased from Life Technology Co. All compounds were dissolved in dimethysulfoxide (DMSO).

### Plant material

*C*. *albida* were collected, identified and deposited similar to our previous reports[2].

### Ethics

No specific permissions were required for the described field studies. The locations are neither privately owned nor protected by the Chinese government. No endangered or protected species were sampled.

### Extraction and Isolation

The plant material (1 kg) was reflux-extracted with 95% EtOH and concentrated under vacuum to produce 120 g of extract. The extract was then partitioned repeatedly with P.E, EtOAc, and *n*-BuOH. The EtOAc layer (25 g) was fractionated using silica gel with P.E./EtOAc (100:1–1:1) as the mobile phase to afford 11 fractions (Fr. 1 to Fr. 11). Fr. 2 was further subjected to successive column chromatography including silica gel (P.E./EtOAc) and Sephadex LH-20 (MeOH:CH_2_CL_2_, 40:60) to afford **5** (12 mg), **6** (8 mg), **9** (10 mg), **10** (9 mg), **11** (8 mg), and **12** (6 mg).

Fr. 3 and Fr. 4 were separated by silica gel and eluted with P.E./EtOAc (100:1–1:1) to afford three fractions (Fr. 3.1 to Fr. 3.3, Fr. 4.1 to Fr. 4.3), respectively. Fr. 3.2 was subjected to preparative HPLC using MeOH/H_2_O (75:25) to give **1** (10 mg), **2** (8 mg), and **3** (12 mg). Fr. 3.3 was chromatographed on a Sephadex LH-20 column using MeOH as the mobile phase to obtain **13** (8 mg) and **14** (9 mg). Similarly, Fr. 4.2 and Fr. 4.3 were subjected to preparative HPLC with MeOH/H_2_O (75:25 and 80:20, respectively) to yield **4** (6 mg), **7** (6 mg), **8** (5 mg), **15** (6 mg), and **16** (5 mg), respectively.

Crotadihydrofuran A (**1**) Yellow oil, C_20_H_18_O_6,_ HRESIMS (*m*/*z*: 377.0993, [M+Na] ^+^, calculated for 377.1001), [α]_D_^20^ +97° (c 0.07, MeOH). ^1^H and ^13^C NMR are in Table 1.

**Table 1:** ^1^H, ^13^C NMR (CD3OD) data of compound 1, 2, 3.^a^

	1^b^		2^c^		3^c^	
	δ_H_	δ_C_	δ_H_	δ_C_	δ_H_	δ_C_
2	4.23, d (12)	75.4	8.14, s	145.8	8.13, s	156.5
	4.84, d (12)					
3	-	75.9	-	144.7	-	115.1
4	-	192.7	-	176.8	-	179.4
4a	-	118.9	-	116.8	-	117.6
5	7.76, d (8.5)	131.2	8.04, d (8.8)	128.1	8.07, d (8.8)	128.5
6	6.51, dd (8.5, 1.5)	112.2	6.93, dd (8.8, 2.2)	117.7	6.93,dd (8.8, 2.2)	116.7
7	-	166.8	-	165.2	-	164.9
8	6.32, d (1.5)	103.8	6.84, d (2.2)	103.3	6.88, d (2.2)	103.1
8a	-	165.0	-	159.7	-	159.7
1′	-	113.9	-	140.3	-	113.8
2′	-	153.3	-	148.3	-	153.8
3′	-	114.8	-	115.7	-	115.1
4′	-	163.2	-	159.2	-	163.2
5′	6.28, d (8.0)	101.9	6.20, d (8.4)	100.8	6.37, d (8.1)	102.3
6′	7.24, d (8.0)	128.4	6.83, d (8.4)	120.3	6.98, d (8.1)	131.9
1″	2.90, dd, (15.5, 8.5)	33.4	2.94, dd, (15.8, 8.0)	33.4	2.94,dd, (15.6, 7.8)	33.3
	3.25, dd, (15.5, 8.5)		3.31, dd, (15.8, 8.0)		3.31,dd, (15.6, 7.8)	
2″	5.15, t (8.5)	87.8	5.18, t (8.8)	87.8	5.23, t (8.8)	87.6
3″	-	146.1	-	146.6	-	145.9
4″	5.03, s	112.4	5.04, s	111.9	5.07, s	112.0
	4.82, s		4.90, s		4.89, s	
5″	1.72, s	17.4	1.75, s	17.2	1.77, s	17.2

^a^ (*J* in Hz); chemical shifts are given in ppm

^b^ 500/125 MHz

^c^ 400/100 MHz.

Crotadihydrofuran B (**2**) White powder, C_20_H_16_O_6,_ HRESIMS, (*m/z*: 353.1028 [M+H]^+^, calculated for 353.1025), [α]_D_^20^ +67° (c 0.07, MeOH). ^1^H and ^13^C NMR are in Table 1.

Crotadihydrofuran C (**3**) Yellow needle, C_20_H_16_O_5,_ HRESIMS (*m/z*: 337.1064, [M+H]^+^, calculated for 337.1076), [α]_D_^20^ + 82° (c 0.07, MeOH). ^1^H and ^13^C NMR are in Table 1.

### X-ray crystallographic analysis[2]

The single-crystal X-ray diffraction data were collected with a Bruker APEX-II CCD area detector diffractometer employing graphite-monochromated CuKα radiation (λ = 1.54178 Å) at 133(2) K. Data collection and integration were performed using a Bruker APEX2 and Bruker SAINT.

Crotadihydrofuran C X-ray crystallography data included: C_21_H_20_O_6_ (MeOH); Mr = 368.37; monoclinic, a = 17.2046(5) Å, b = 6.8904(2) Å, c = 15.0794(4) Å; α = 90°, β = 103.962(2°, λ = 90°; V = 1734.80(8) Å^3^; Z = 4; Dcalc = 1.410 Mg/m^3^; F(000) = 776; μ(CuKα) = 0.859 mm^-1^; crystal dimensions 0.30 × 0.12 × 0.04 mm; θ range of 3.02–64.95°; independent reflections: 2571 (R(int) = 0.0565). The final R1 values were 0.0383; wR2 = 0.1048 [I > 2σ(I)]. Absolute structure parameter: 0.0(2). CCDC number: 885689.

### 3T3-L1 pre-adipocytes differentiation

The 3T3-L1 preadipocyte cell line was cultured and differentiated as previously described[15, 20]. Briefly, pre-adipocytes were inoculated into 12- (2 × 10^5^) or 24- (1 × 10^5^) well plates and incubated in DMEM (Hyclone, Logan, UT) supplemented with 10% FBS. These were grown to full confluence over 2 days. The cells were differentiated in initiation medium (DMEM supplemented with 10% FBS) supplemented with 10 μg/ml insulin (Sigma-Aldrich. St. Louis, MO), 1 μM dexamethasone (DEX; Sigma-Aldrich. St. Louis,MO) and 10 μM Rosiglitazone (ROS; Sigma-Aldrich. St. Louis,MO) (Day 0)after confluence. After 2 days of induction, the medium was changed with only insulin in DMEM with 10% FBS for an additional 2 days. During the induction, **1**, **2**, and **3** (indicated concentration) were reconstituted in DMSO and added to the medium at the beginning of induction of 3T3-L1 cells[20]. For RT-PCR experiment, cells were treated with compounds (50 μM) and differentiation medium after confluence for 2 days and 8 days[20]. The cells were incubated at 37°C, in a 7.5% CO_2_ incubator throughout the experiment.

### Proliferation assay

Cell viability was investigated with the MTT [3-(4.5-dimethylthiazol-2-yl)- 2,5-diphenyl-tetrazolium bromide] assay. Briefly, 1 × 10^4^ cells were seeded into 96-well plates and cultured in DMEM supplemented with 10% FBS and then incubated with compound 1, 2, and 3 (0, 12.5, 25, 50, 100 μM) for 24 or 48 h. After treatment, MTT was added to each well and incubated at 37 C. After 4 h, the medium was removed and then blue formazan crystal was dissolved in 100μL DMSO. Absorbance values were collected at 570 nm using a fluorescent plate reader. The data were presented as percent cell viability versus control group. In each experiment, proliferation was determined in six replicate wells, and the overall experiment was repeated at least three times.

### Oil red O staining

Eleven days after the induction of differentiation, the cells were washed with phosphate-buffered saline (PBS) twice, fixed in fresh 10% formalin for 10 min at room temperature, and stained with Oil Red O (Sigma, St. Louis, MO) at 60°C for 30 min. The cells were washed once with PBS and then photographed[15].

### Transfection of cultured cells and luciferase assays[15, 21]

HEK 293T cells were inoculated into a 48-well culture plate at 5 × 10^4^ cells/well and incubated in 5% CO_2_ at 37°C with DMEM and 10% FBS overnight. The expression plasmid pCMX-Gal-mPPAR-γ-LBD, the Gal4 reporter vector MH100×4-TK-Luc and Renilla-Luc were gifts from Dr. C. Huang. The reporter assay was conducted as described previously[21]. The transfection mixture contained total plasmids, and FuGENE HD (Roche, Germany) and was added to 293T cells overnight and the removed. The solution was then changed to fresh media containing PPAR-γ agonist rosiglitazone or the compounds of interest at the previously determined concentration. All measurements were performed according to the protocol of the Dual-Luciferase Reporter Assay System (Promega, Madison, WI, USA). Luciferase activity was measured and displayed as values ± SE, which was corrected for transfection efficiency using the renilla luciferase activity. All transfection experiments were achieved in triplicate and repeated three times independently[21].

### Quantitative real-time PCR[20]

Total RNA from 3T3-L1 cells was extracted with a spin column (Qiagen, Hilden, Germany) according to the manufacturer’s instructions. The first-strand cDNA was synthesized from 3μg of total RNA using a cDNA kit (Fermentas, Madison, WI, USA). The RNA expression levels were then quantified with a quantitative real-time RT-PCR using SYBR Green PCR Master Mix (Applied Biosystems, USA) and ABI Step One Plus Real Time PCR system (Applied Biosystems, USA)[21]. The forward and reverse primer sequences used in the RT-PCR were given in S1 Table. The results were calculated relative to β-actin.

### Statistical analysis

The data are the mean ± SE. Significant differences between means were evaluated via one-way analysis of variance (ANOVA) and a two-tailed unpaired Student’s test and results were considered significant when p < 0.05.

## Results and Discussion

**Fig 1:**
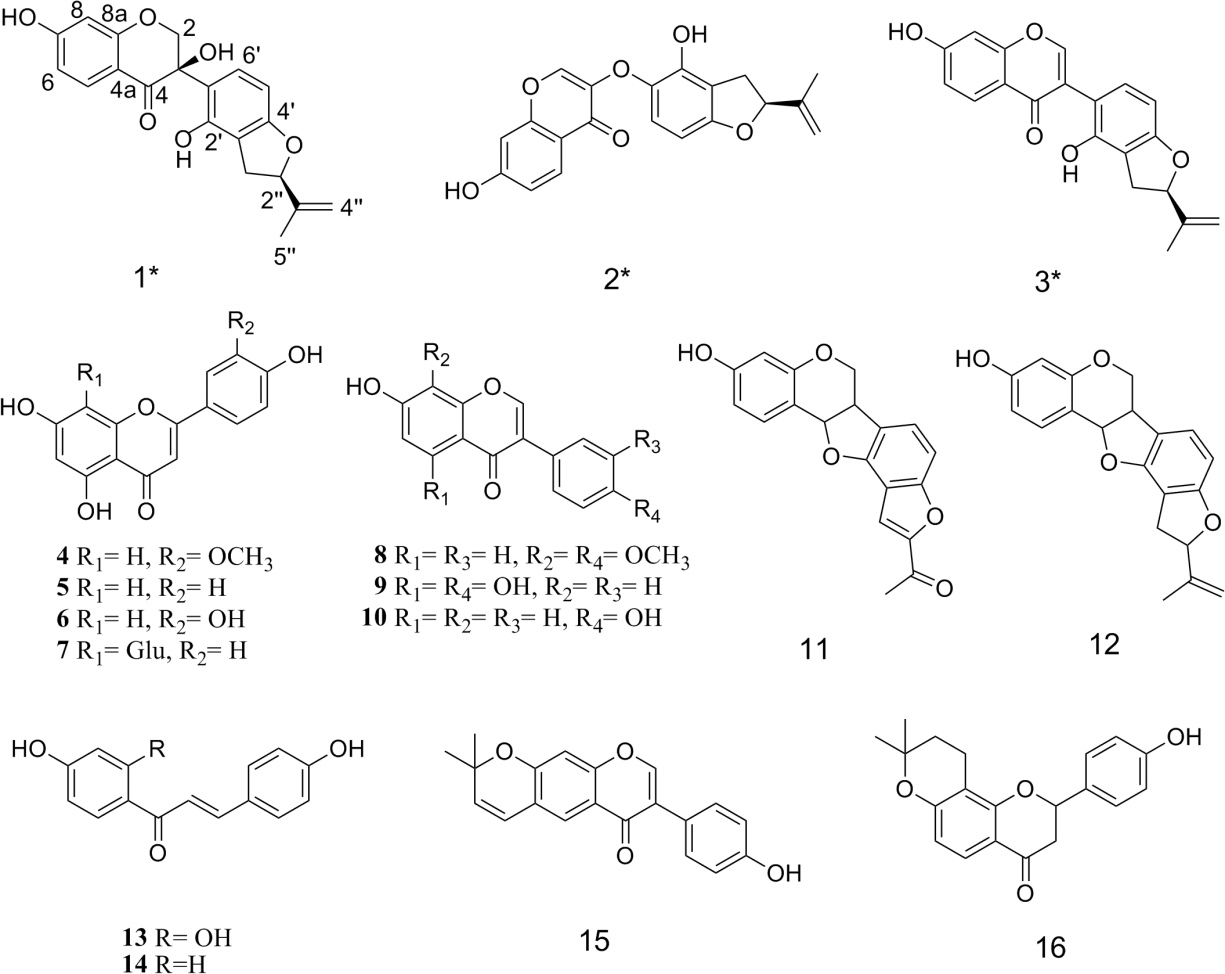
Structures of compounds 1–16 isolated from *C*. *albida*. Asterisks indicate new compounds.

Crotadihydrofuran A (**1**) was obtained as a yellow oil. Its molecular formula was determined as C_20_H_18_O_6_ by HRESIMS (*m*/*z*: 377.0993, [M+Na] ^+^, calculated for 377.1001; S1 Fig), [α]_D_^20^ +97° (c 0.07, MeOH). The ^1^H NMR spectrum of **1** (Table 1; S2 Fig) shows two one-proton doublets at δ_H_ 4.84 (H-2a) and 4.23 (H-2b) (each *J =* 12.0 Hz) as well as a typical ABX aromatic proton system at δ_H_ 7.76 (1H, d, J = 8.5 Hz, H-5), 6.51 (1H, dd, J = 8.5, 1.5 Hz, H-6) and 6.32 (1H, d, J = 1.5 Hz, H-8). There were two *ortho*-coupled doublets at δ_H_ 6.28 (1H, d, *J =* 8.0 Hz, H-5′) and δ_H_ 7.24 (1H, d, *J =* 8.0 Hz, H-6′). The ^13^C NMR data of **1** (Table 1; S3 Fig) showed one methylene carbon signal at δ_C_ 75.4(C-2), one quaternary carbon signal at δ_C_ 75.9 (C-3), and a carbonyl signal at δ_C_ 192.7 (C-4). A 3-hydroxyisoflavanone skeleton was seen in **1**[22]. Furthermore, the ^1^H NMR spectrum of **1** also exhibited the presence of a 2″-isopropenyl dihydrofuran ring—a methyl group singlet signal at δ_H_ 1.72 (3H, s, H-5″) and two broad singlets for an exomethylene group signal at δ_H_ 4.82 (1H, s, Ha-4″) and 5.03 (1H, s, Hb-4″). This suggests the presence of an isopropenyl side chain. There was also an endocyclic methylene group signal at δ_H_ 2.90 (1H, dd, J = 15.5, 8.5, Ha-1″) and 3.25 (1H, J = 15.5, 8.5, Hb-1″) as well as a triplet signal at δ_H_ 5.15 (1H, t, J = 8.5, H-2″) for methane. These were characteristic of a dihydrofuran ring[23] that was substituted at position 2″. The location of the 2″-isopropenyl dihydrofuran unit on ring B was determined based on the HMBC (S2 Table; S4 and S5 Figs) correlations from δ_H_ 2.90 and 3.25 (Ha-1″ and Hb-1″) to δ_C_ 153.3 (C-2′), 114.8 (C-3′), and 163.2 (C-4′). Moreover, the R configuration of C-3 was determined based on its circular dichroism (CD) spectrum (S6 Fig), which gave a positive effect at 334 nm[22, 24]. In the NOESY spectrum (S2 Table; S7 and S8 Figs), H-2″ (δ_H_ 5.15) correlated with Hb-1″ (δ_H_ 3.25) but not with Ha-1″ (δ_H_ 2.90); H-5″ (δ_H_ 1.72) correlated with Ha-1″ (δ_H_ 2.90) but not with Hb-1″ (δ_H_ 3.25). This indicated that H-2″had an α-orientation. Thus, the structure of crotadihydrofuran A (**1**) was determined as 3*R*-3, 7, 2′- trihydroxy-2″α-isopropenyl dihydrofuran [6″, 7″: 3′, 4′]-isoflavanone (Fig 1).

Crotadihydrofuran B (**2**) had a molecular formula of C_20_H_16_O_6_ based on its HRESIMS, (*m/z*: 353.1028 [M+H]^+^, calculated for 353.1025; S10 Fig). It was a white powder with [α]_D_^20^ +67° (c 0.07, MeOH). The ^1^H and ^13^C NMR of **2** (Table 1; S11 and S12 Figs) showed an α, β-unsaturated carbonyl carbon resonance at δ*c* 176.8 (C-4), an olefinic singlet proton signal at δ_H_ 8.14 (1H, s, H-2) with a corresponding carbon atom signal at δ_C_ 145.8 (C-2), as well as a typical ABX aromatic proton system at δ_H_ 8.04 (1H, d, J = 8.8 Hz, H-5), δ_H_ 6.93 (1H, dd, J = 8.8, 2.2 Hz, H-6), and δ_H_ 6.84 (1H, d, J = 2.2 Hz, H-8)—these indicated a characteristic presence on the 2, 7-dioxygenated chromone nucleus. In addition, the ^1^H and ^13^C NMR spectra data of **2** had a 2″-isopropenyl dihydrofuran unit [C-1″ (δ_C_ 33.4), C-2″ (δ_C_ 87.8), C-3″ (δ_C_ 146.6), C-4″ (δ_C_ 111.9), C-5″ (δ_C_ 17.2)], and two *ortho*-coupled doublets from the AM system at δ_H_ 6.20 (1H, d, *J =* 8.4 Hz, H-5′) and δ_H_ 6.83 (1H, d, *J =* 8.4 Hz, H-6′). (Table 1; S11 and S12 Figs). The ^1^H and ^13^C NMR data of **2** also closely resembled those of **3**. The only significant difference in their ^13^C NMR spectra were of the carbon signals at δc 115.1 (C-3) and 113.8 (C-1′) in **3** shifted to δc 144.7 (C-3) and 140.3 (C-1′) in **2**. These were due to an electron-withdrawing effect of the oxygen atom at C-3. Comparing their molecular formula and weight gives further evidence for this assumption **2** has one more oxygen atom than **3**. Thus, ring B was connected to ring C by an oxo-bridge. The configuration of H-2″ was the same α-orientation as **1** via similar NOESY correlations (S2 Table; S15 and S16 Figs). On the basis of this evidence, the structure of crotadihydrofuran B (**2**) was determined as 7-hydroxy-3-(2′-hydroxy-2″α-isopropenyl-dihydrobenzofuran-1′-oxy)-chromen-4-one (Fig 1).

Correspondingly, the unusual new structure **2** that is unprecedented in the natural isoflavonoid derivatives might biosynthetically be derived from rotenoid[25] (S18 Fig). We assumed that intermediates (i, ii, and iii) were obtained via hydrolysis of the ring, oxidation, loss of carboxyl group, and elimination of hydrogen.

**Fig 2:**
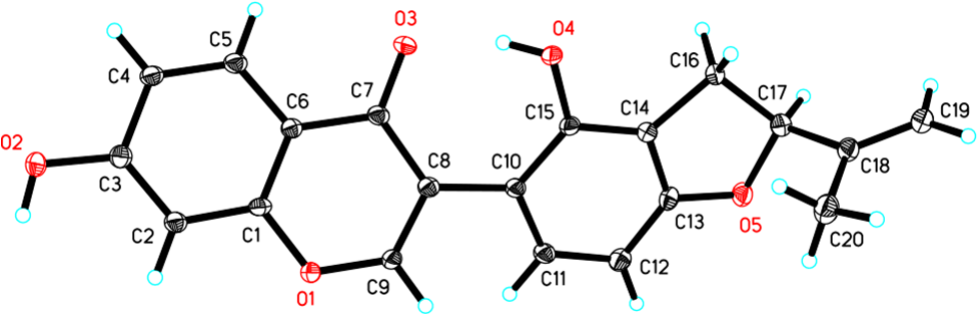
ORTEP Drawing (X-ray Analysis, CuKα) of 3.

Crotadihydrofuran C (**3**) was isolated as a yellow needle with an [α]_D_^20^ + 82° (c 0.07, MeOH) and a the molecular formula of C_20_H_16_O_5_ deduced from HRESIMS (*m/z*: 337.1064, [M+H]^+^, calculated for 337.1076; S19 Fig). The NMR spectra of **3** showed a singlet resonance at δ_H_ 8.13 and corresponding olefinic oxymethine signal at δ_C_ 156.5 (Table 1; S20 and S21 Figs) due to H-2 and C-2, respectively. There was an α, β-unsaturated carbonyl carbon resonance at δ*c* 179.4 (C-4) suggesting that this compound has an isoflavone skeleton[26]. Similar to **1**, the ^1^H and ^13^C NMR spectra data of **3** also showed a 2″-isopropenyl dihydrofuran unit, an ABX system, and two *ortho*-coupled doublets (Table 1). The configuration of H-2″ was the same α-orientation as **1** as seen in their similar NOESY correlations (S2 Table; S24 and S25 Figs). Therefore, the crotadihydrofuran C (**3**) was determined to be 7, 2′- dihydroxy-2″ α-isopropenyl dihydrofuran [6″, 7″: 3′, 4′]-isoflavone. The X-ray diffraction (CuKα) of **3** further confirmed this structure, as C-2″ S (Fig 2). On comparison with the configuration of **3**, the absolute configuration of C-2″ in **1** and **2** were also determined to be S (Fig 1).

Thirteen known cases have been identified through comparison of the NMR data to reported compounds such as chrysoeriol (**4**)[27], apigenin (**5**)[28], digitoflavone(**6**)[28], vitexin(**7**)[29], isoafrormosin (**8**)[30], genisteol (**9**)[31], daidzein (**10**)[32], crotafuran B (**11**)[33], barbacarpan (**12**)[23], isoliquiritigenin (**13**)[34], 4', 4- dihydroxychalcone (**14**)[35], alpinumisoflavone (**15**)[36] and 4'-hydroxyIsolonchocarpin (**16**)[37] (Fig 1).

We assayed whether compounds (1–16) isolated from *C*. *albida* could modulate transactivities of PPAR-γ on the Dual-Luciferase Reporter Assay System, because several studies have reported that regulation of PPAR-γ is key to the biological effects of flavones. The results showed that **1**, **2**, and **3** (crotadihydrofuran A, B, C) were potent suppressors of PPAR-γ (Table 2). Compounds **1**, **2**, and **3** are undescribed compounds, and the strengths of inhibition for transactivity of PPAR-γ are the strongest among all compounds. Therefore, we focused on **1**, **2**, and **3**.

**Table 2:** Effects of Compounds on Transcription Activity of Increased PPAR-γ.^a^

Compounds	Action ^b^ ^c^
1, 2, 3	-
5, 6, 9, 10, 13, 14	+
4, 7, 8, 11, 12, 15, 16	±

^a^ Increased on Rosiglitazone.

^b^ +: promotion;-: inhibition; ±: no effect.

^c^ The other compounds were inactive at 50 μM.

**Fig 3:**
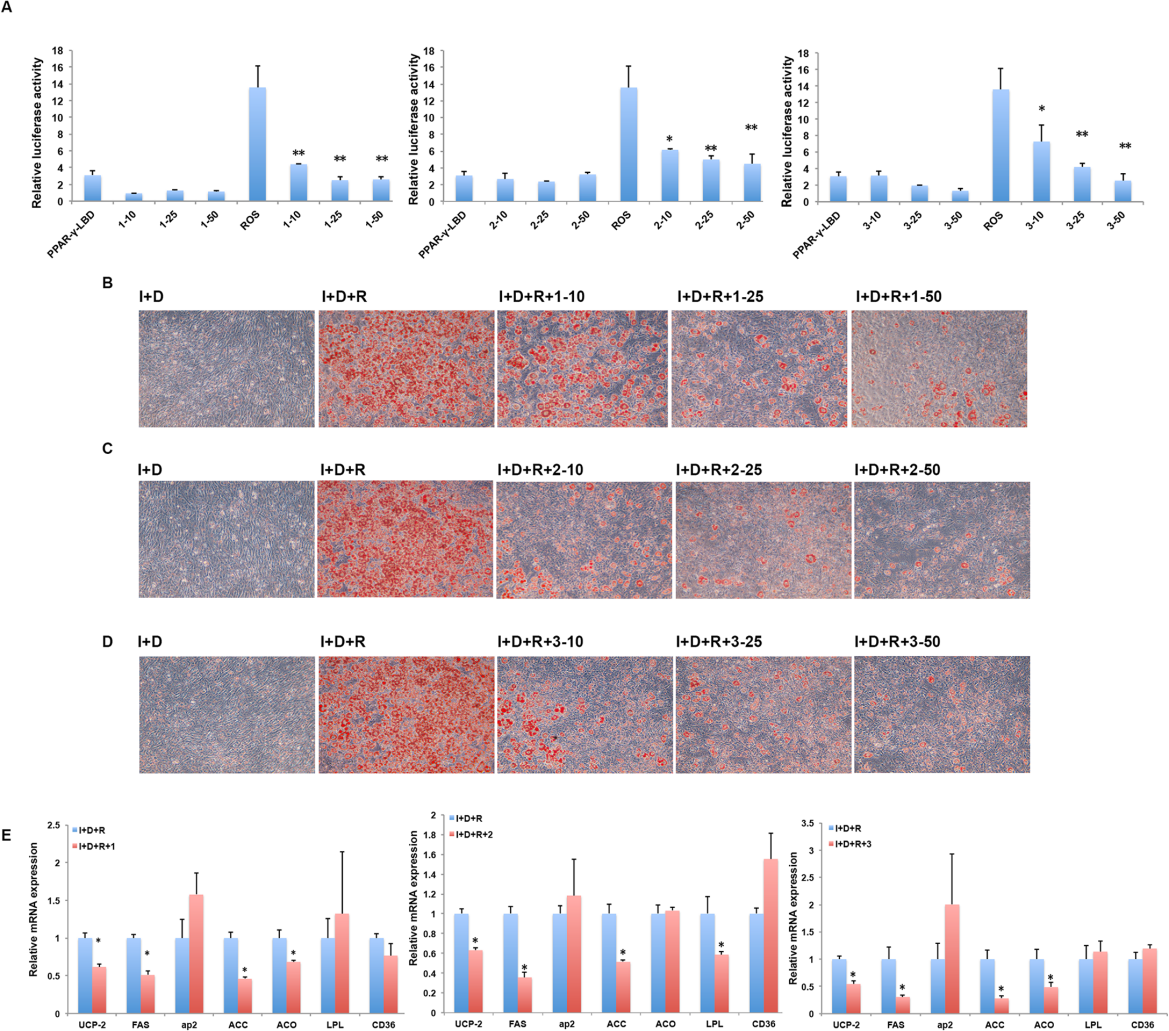
Compounds inhibit 3T3-L1 adipocyte differentiation and adipogenesis related gene expression by inhibiting transcription activity of PPAR-γ. (A) Compounds inhibit the transcription activity of PPAR-γ. (B) Compound 1 suppresses 3T3-L1 adipocyte differentiation induced by differentiation medium. (C) Compound 2 suppresses 3T3-L1 adipocyte differentiation induced by differentiation medium. (D) Compound 3 suppresses 3T3-L1 adipocyte differentiation induced by differentiation medium. Differentiation medium includes 10 μg/ml insulin, 1 μM dexamethasone, and 10 μM rosiglitazone. Oil red O staining of 3T3-L1 cells was performed on day 10. Compounds were added to the medium at the beginning of induction of 3T3-L1 cells at the indicated concentration. (E) Real-time RT-PCR results of gene expression levels at day 10 in 3T3-L1 adipocyte. Cells were treated with compounds (50 μM) and differentiated for 10 days. Control: growth medium. I+D: insulin and dexamethasone. I+D+R: insulin, dexamethasone, and rosiglitazone. Mouse beta-actin was used as the control, and values representing mRNA of the untreated cells were defined as 1. Data are presented as means ± SE (n = 3). *P < 0.05, **P < 0.01.

The results indicated that the luciferase activity increased 4-fold when the PPAR-γ agonist rosiglitazone was used. Compounds **1**, **2**, and **3** also significantly inhibited the rosiglitazone-stimulated PPAR-γ transactivity in a dose-dependent manner (Fig 3A). At 50 μM, transactivity of PPAR-γ induced by rosiglitazone was inhibited by **1** up to 64%, **2** up to 80%, and **3** up to 85%. These data suggest that **1**, **2**, and **3** inhibit PPAR-γ transactivity and may be an antagonist of PPAR-γ.

Based on their inhibition of PPAR-γ transactivity, we postulated that **1**, **2**, and **3** might suppress adipocyte differentiation via direct targeting of PPAR-γ activity. To test this hypothesis, we used a 3T3-L1 adipocyte differentiation model[20]. Rosiglitazone could strongly promote 3T3-L1 adipocyte differentiation in the presence of insulin and dexamethasone in the culture medium. During the induction, the compounds (10, 25 and 50 μM) were added to the medium at day 0, and we observed their effects on 3T3-L1 adipocyte differentiation at day 10[15]. As shown in Fig 3B, **1** inhibited the most adipocyte differentiation at doses of 50 μM and the effect of inhibition weakened as concentration decreased. Similar effects were seen in 3T3-L1 adipocytes treated with **2** and **3** (Fig 3C and 3D). The cell proliferation assay demonstrated that the suppression of adipocyte differentiation by isolated compounds was not due to cytotoxicity because compound treatment did not influence the cell viability and proliferation (S27 Fig). These findings suggest that three novel isoflavonoids inhibit the 3T3-L1 differentiation and adipogenesis by potentially suppressing PPAR-γ activity.

Activation of PPAR-γ triggers the expression of various genes, containing uncoupling protein 2 (UCP-2), fatty acid synthase (FAS), adipose fatty acid-binding protein (aP2), acetyl coenzyme A carboxylase (ACC), acyl-CoA oxidase (ACO), lipoprotein lipase (LPL) and cluster of differentiation 36 (CD36)[21]. These are closely related to lipogenesis, fatty acid synthesis, and energy metabolism. It is conceivable that the compounds could inhibit the target genes expression of PPAR-γ, because **1**, **2**, and **3** inhibited the PPAR-γ transactivity and blocked adipocyte lipid accumulation. We tested the effects of **1**, **2**, and **3** on mRNA expression levels in 3T3-L1 cells. In 3T3-L1 cells, **1** remarkably reduced mRNA expression of UCP-2, FAS, aP2, ACC and CD36, **2** significantly inhibited mRNA levels of FAS, aP2, ACC, LPL and CD36 and **3** decreased mRNA expression of UCP-2, FAS, ACC and ACO (Fig 3E). Collectively, the results suggest that **1**, **2**, and **3** affect adipogenesis and lipid accumulation by modulating PPAR-γ signaling.

## Supporting Information

S1 Fig HRESIMS spectrum of 1. (TIF)

S2 Fig ^1^H-NMR spectrum (500 MHz, CD_3_OD) of 1. (TIF)

S3 Fig ^13^C-NMR spectrum (125 MHz, CD_3_OD) of 1. (TIF)

S4 Fig HMBC(1) spectrum of 1. (TIF)

S5 Fig HMBC(2) spectrum of 1. (TIF)

S6 Fig Experimental CD spectrum of 1. (TIF)

S7 Fig NOESY(1) spectrum of 1. (TIF)

S8 Fig NOESY(2) spectrum of 1. (TIF)

S9 Fig HSQC spectrum of 1. (TIF)

S10 Fig HRESIMS spectrum of 2. (TIF)

S11 Fig ^1^H-NMR spectrum (400 MHz, CD_3_OD) of 2. (TIF)

S12 Fig ^13^C-NMR spectrum (100 MHz, CD_3_OD) of 2. (TIF)

S13 Fig HMBC(1) spectrum of 2. (TIF)

S14 Fig HMBC(2) spectrum of 2. (TIF)

S15 Fig NOESY(1) spectrum of 2. (TIF)

S16 Fig NOESY(2) spectrum of 2. (TIF)

S17 Fig HSQC spectrum of 2. (TIF)

S18 Fig Hypothetical Biogenetic Pathway of 2. (TIF)

S19 Fig HRESIMS spectrum of 3. (TIF)

S20 Fig ^1^H-NMR spectrum (400 MHz, CD_3_OD) of 3. (TIF)

S21 Fig ^13^C-NMR spectrum (100 MHz, CD_3_OD) of 3. (TIF)

S22 Fig HMBC(1) spectrum of 3. (TIF)

S23 Fig HMBC(2) spectrum of 3. (TIF)

S24 Fig NOESY(1) spectrum of 3. (TIF)

S25 Fig NOESY(2) spectrum of 3. (TIF)

S26 Fig HSQC spectrum of 3. (TIF)

S27 Fig Effect of compounds on proliferation of 3T3-L1 cells in vitro. (A) Compound 1. (B) Compound 2. (C) Compound 3. After 24 and 48 hours, proliferation was assessed by MTT. The cell viability was shown as inhibitory ratio (% of control). Data are presented as means ± SE (n = 6). *P < 0.05, **P < 0.01.
(TIF)

S1 Table Sequences of the primers used in real time PCR (Mus musculus). (TIF)

S2 Table HMBC, NOESY(CD_3_OD) data of compound 1, 2, 3. (TIF)
