# Supplementary figures and images for: Isoflavonoids from Crotalaria albida Inhibit Adipocyte Differentiation and Lipid Accumulation in 3T3-L1 Cells via Suppression of PPAR-γ Pathway
Source: PLoS One. 2015 Aug 18;10(8):e0135893. doi: 10.1371/journal.pone.0135893 (PMC4540591; doi:10.1371/journal.pone.0135893)

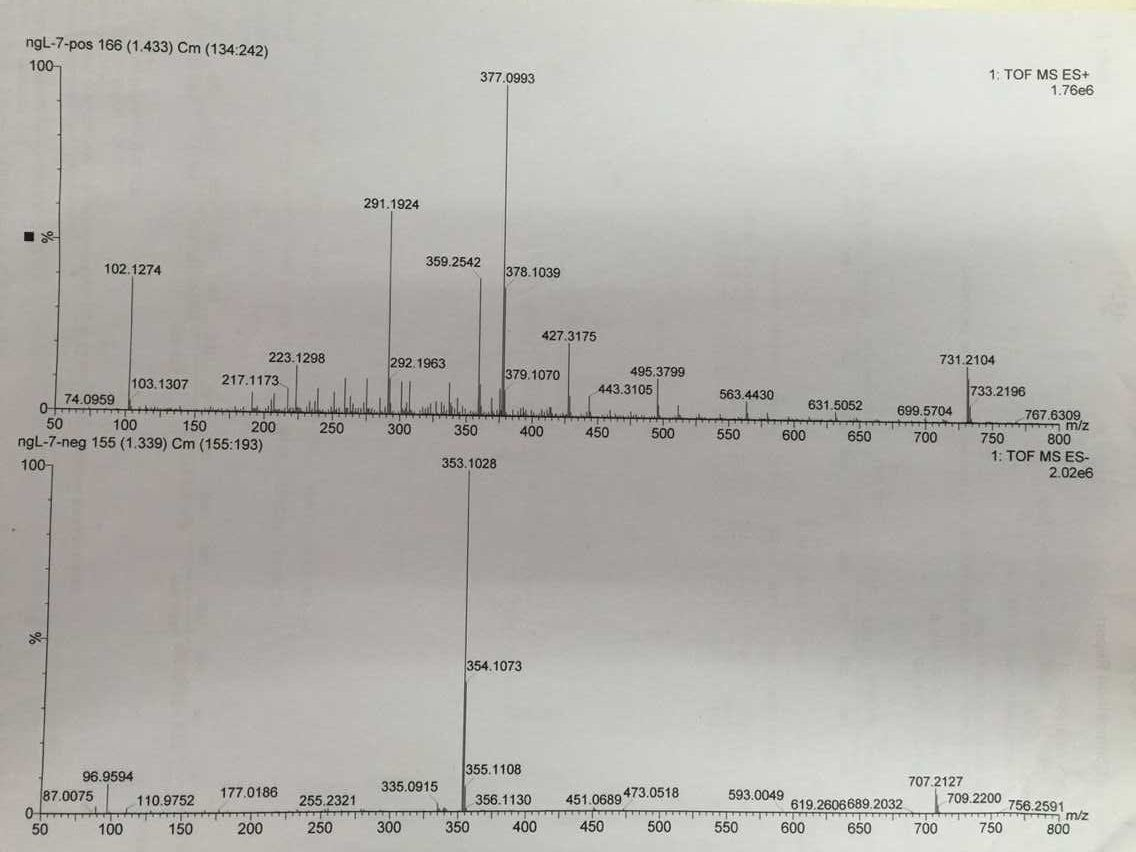

Supplement: S1 Fig — (TIF) [file pone.0135893.s001.tif]

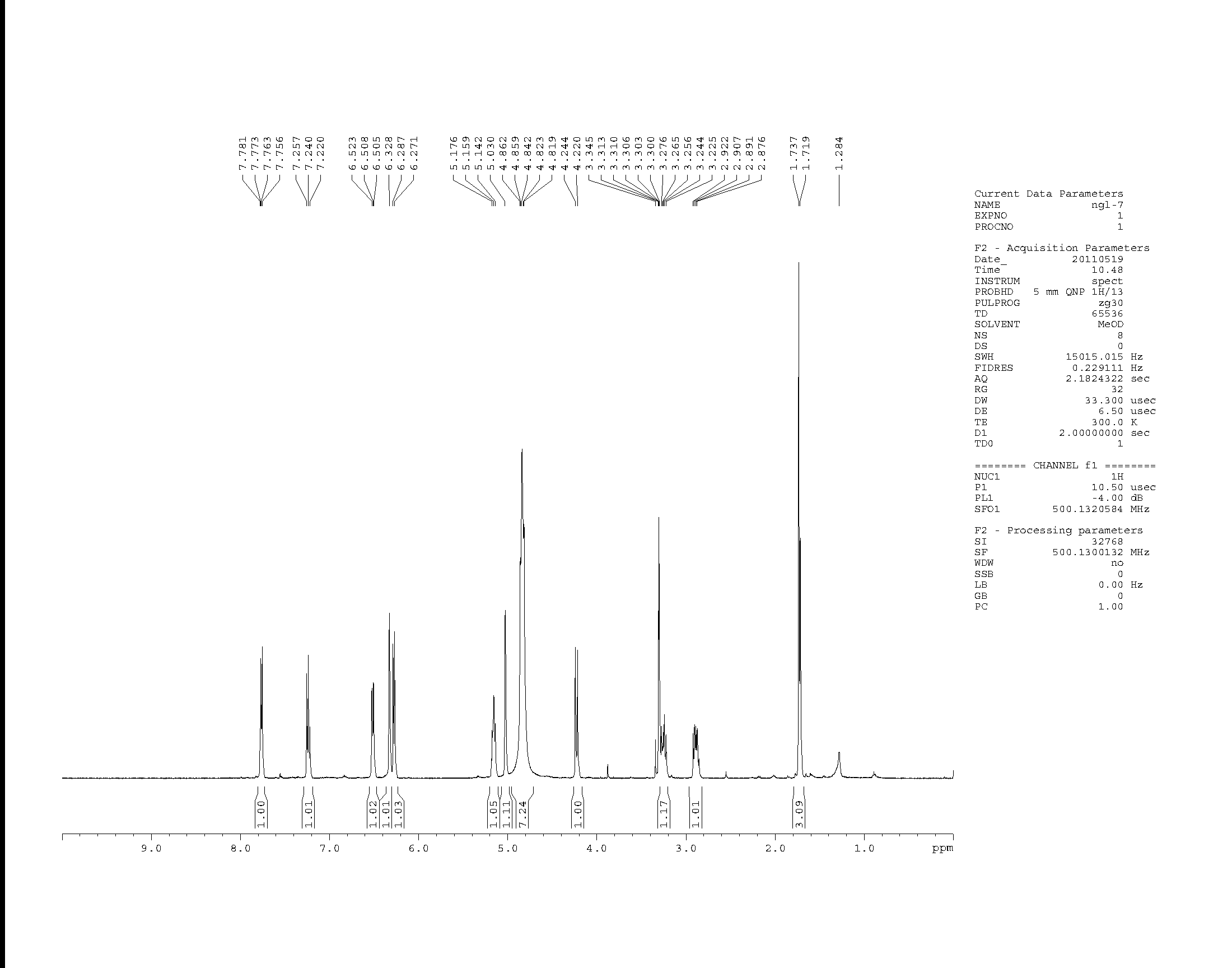

Supplement: S2 Fig — (TIF) [file pone.0135893.s002.tif]

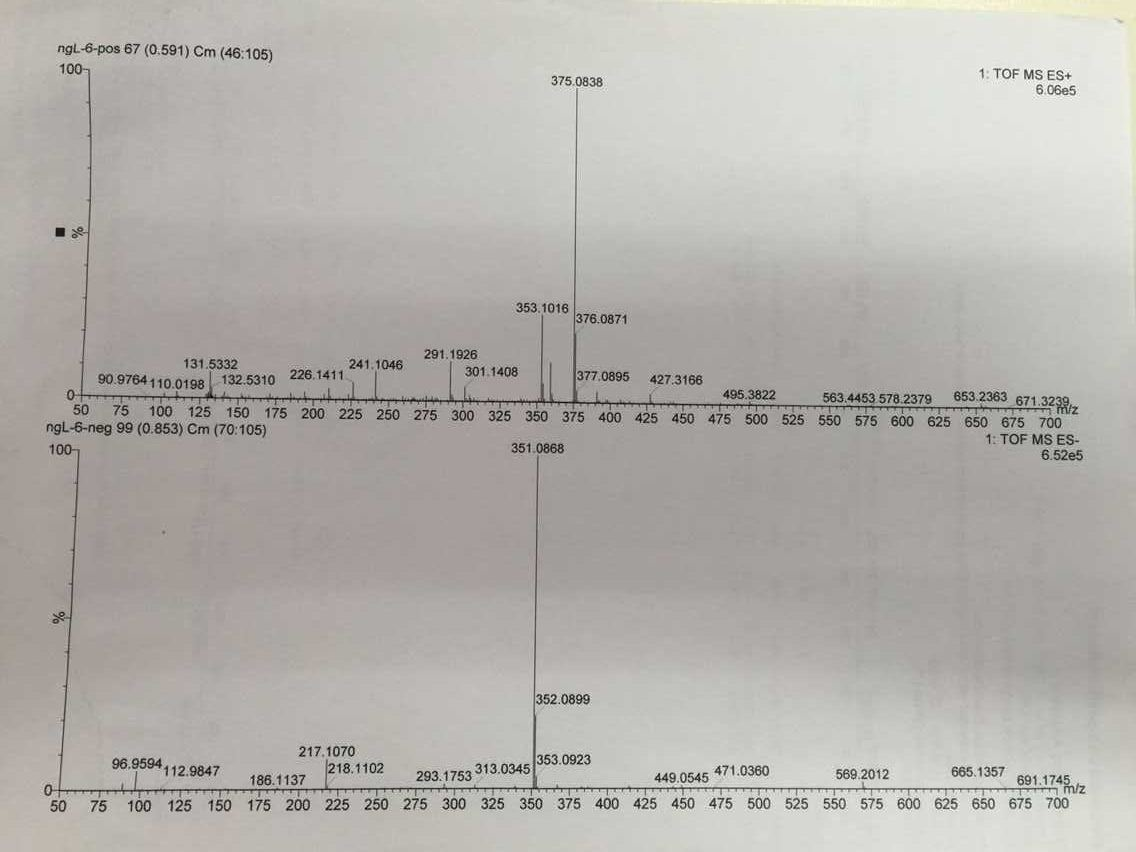

Supplement: S10 Fig — (TIF) [file pone.0135893.s010.tif]

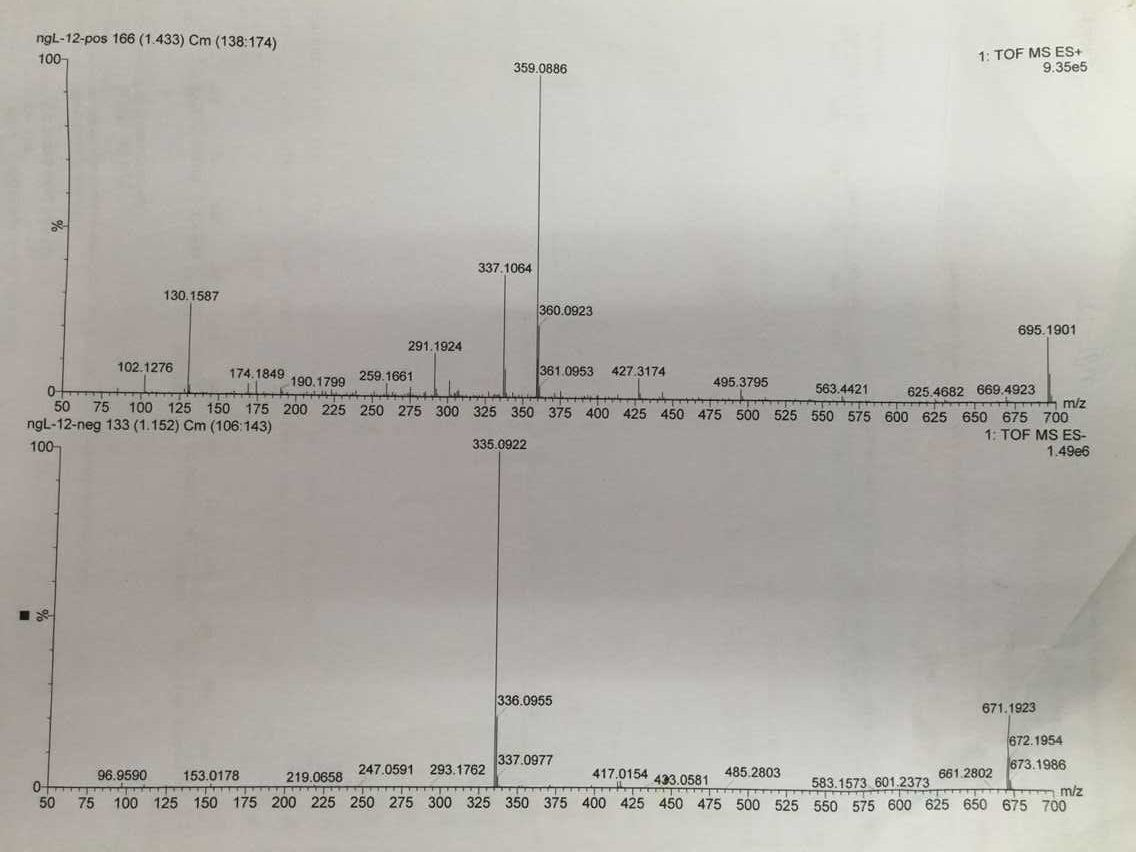

Supplement: S19 Fig — (TIF) [file pone.0135893.s019.tif]

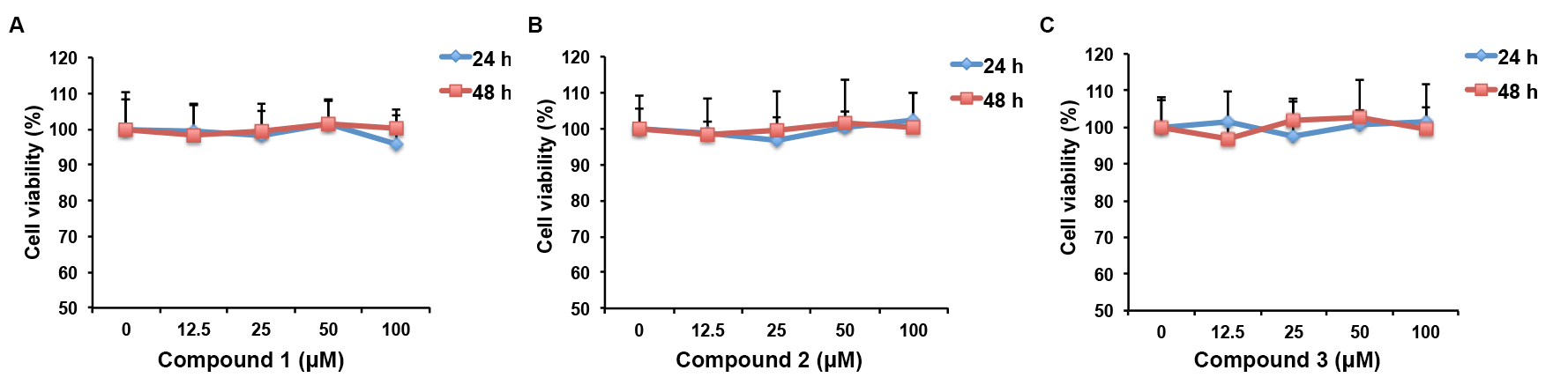

Supplement: S27 Fig — (A) Compound 1. (B) Compound 2. (C) Compound 3. After 24 and 48 hours, proliferation was assessed by MTT. The cell viability was shown as inhibitory ratio (% of control). Data are presented as means ± SE (n = 6). *P < 0.05, **P < 0.01. (TIF) [file pone.0135893.s027.tif]
